# Immune-metabolic marker of albumin-to-fibrinogen ratio based prognostic nomogram for patients following peritoneal dialysis

**DOI:** 10.3389/fmed.2024.1462874

**Published:** 2024-08-30

**Authors:** Xiao-wen Ye, Yun-xia Shao, Ying-chun Tang, Xiong-jun Dong, Ya-ning Zhu

**Affiliations:** Department of Nephrology, Wuhu Hospital, East China Normal University, Wuhu, China

**Keywords:** peritoneal dialysis, albumin-to-fibrinogen ratio, immune-metabolic biomarker, nomogram, prognosis

## Abstract

**Background:**

The nutritional status and coagulation function of peritoneal dialysis (PD) patients are closely associated with their prognosis. This study aims to investigate the prognostic value of the albumin-to-fibrinogen ratio (AFR) on mortality in PD patients and to establish a prognostic prediction model based on AFR.

**Methods:**

We retrospectively collected data from 148 PD patients treated at our hospital between Oct. 2011 and Dec. 2021. Using the “survminer” package in R, we determined the optimal cutoff value for AFR and divided the patients into low-AFR and high-AFR groups. The primary endpoint of this study was overall survival (OS). Univariate and multivariate Cox analyses were used to assess the impact of AFR and other factors on prognosis, and a corresponding prognostic prediction model was constructed using a nomogram, which was evaluated through ROC curves, the c-index, and calibration plots.

**Results:**

The optimal cutoff value for AFR was 9.06. In the entire cohort, 30 patients (20.2%) were classified into the low-AFR group. Compared to the high-AFR group, patients in the low-AFR group were older, had lower total urine output over 24 h, higher blood urea nitrogen, higher total protein and urinary microalbumin levels, and longer remission times (*p* < 0.05). They also had a poorer OS (HR: 1.824, 95%CI: 1.282–2.594, *p* < 0.05). Multivariate Cox analysis indicated that AFR was an independent prognostic factor for OS (HR: 1.824, 95% CI: 1.282–2.594, *p* < 0.05). A prognostic prediction model based on AFR, age, and cause of ESRD was successfully validated for predicting OS in PD patients.

**Conclusion:**

AFR represents a potential prognostic biomarker for PD patients. The prognostic prediction model based on AFR can provide accurate OS predictions for PD patients, aiding clinicians in making better-informed decisions.

## Introduction

It is estimated that over 272,000 patients worldwide are currently undergoing peritoneal dialysis (PD), accounting for approximately 11% of all dialysis patients globally (1). Dialysis, as a renal replacement therapy, primarily includes hemodialysis (HD) and peritoneal dialysis. Due to its lower operational site requirements and relative cost-effectiveness, PD has become the preferred alternative treatment modality for patients with chronic kidney disease (CKD) (2). The principle of PD involves the exchange of solutes and fluids between the blood in the peritoneal capillaries and the dialysis fluid, with adjustable flow rates to maximize clearance (3, 4). In Asia, the utilization rate of PD varies from 3 to 73%, with China showing particularly significant rates (5, 6).

Over the last decade, there has been a significant increase in the utilization of peritoneal dialysis (PD) in China. This surge can be attributed to several factors, including advancements in PD technology, improved patient education, and supportive healthcare policies. This increase has prompted an intensified focus on understanding the factors that influence the prognosis of PD patients, given the complexities involved in managing chronic kidney disease (CKD) through PD. PD offers numerous advantages over hemodialysis (HD), including greater flexibility, a better quality of life, and lower healthcare costs, making it a vital option for many CKD patients. Despite these benefits, the long-term success of PD depends on multiple factors, including the patient’s nutritional status, inflammation levels, and the presence of comorbidities.

In recent years, serum nutritional indicators have gained increasing attention in cancer research due to their significant impact on patient outcomes. Malnutrition is common among cancer patients and is associated with increased morbidity and mortality (7). Similarly, malnutrition is prevalent among CKD patients, particularly those undergoing PD (8). Malnutrition in PD patients is often multifactorial, resulting from inadequate dietary intake, protein loss through dialysis, and chronic inflammation. Recent studies suggest that hypoalbuminemia, negatively correlated with patient prognosis, may be more attributable to inflammation rather than malnutrition. Serum albumin and prealbumin levels are common indicators of nutritional status. Albumin, synthesized and secreted by the liver, typically constitutes more than 50% of blood proteins, reflecting the protein status of the blood and viscera. Serum albumin (Alb) is commonly used to assess nutritional status (9).

Fibrinogen, a glycoprotein synthesized by the liver, responds to inflammation or activation of the coagulation system, playing a crucial role in the coagulation cascade. Disorders of coagulation, anticoagulation, and fibrinolysis in CKD patients often accompany abnormal coagulation and elevated fibrinogen levels (10, 11). Elevated fibrinogen levels are indicative of an inflammatory state and are associated with a higher risk of cardiovascular events and mortality. However, not all PD patients concurrently exhibit nutritional and coagulation disorders. This variability suggests the need for a more comprehensive biomarker that can simultaneously reflect nutritional and inflammatory status.

Recently, the albumin-to-fibrinogen ratio (AFR) has been introduced as a novel combined biomarker, demonstrating higher prognostic value in certain malignancies such as breast cancer, bladder cancer, and gastric cancer (12). The AFR combines the prognostic information of both albumin and fibrinogen, providing a more integrated view of a patient’s nutritional and inflammatory status. However, there are few reports on its use in benign diseases, especially in PD patients. Given the unique pathophysiological changes in PD patients, including protein loss and chronic inflammation, AFR may offer valuable prognostic insights. This study aims to investigate the impact of AFR and clinically relevant factors on the long-term prognosis of PD patients and to establish and validate a new nomogram for individual risk assessment of OS in PD patients based on AFR. By integrating AFR into a prognostic model, we hope to enhance the accuracy of survival predictions and provide a useful tool for clinicians in the management of PD patients.

## Methods

### Ethics approval and consent to participate

The study was conducted in accordance with the Declaration of Helsinki (as revised in 2013) and approved by the Ethics Committee of Wuhu Hospital Affiliated to East China Normal University (No. 2021-D.-301). Due to the retrospective nature of the study, participant informed consent was waived, and the study design was approved by the appropriate ethics review board.

### Patients and clinicopathological factors

#### Patient selection

Our study retrospectively analyzed the data of PD patients in our hospital between Oct. 2011 and Dec. 2021 and screened out148 eligible patients for further analysis according to the inclusion and exclusion criteria. The authors are accountable for all aspects of the work in ensuring that questions related to the accuracy or integrity of any part of the work are appropriately investigated and resolved.

#### Inclusion and exclusion criteria

Inclusion criteria: (1) Patients aged 18 years and above; (2) PD duration of no less than 3 months; and (3) Patients in generally good health and stable mental status.

Exclusion criteria: (1) History of kidney transplantation or long-term hemodialysis; (2) Recent active infection; (3) Severe liver dysfunction; (4) Active rheumatic diseases, hematological diseases, or malignancies; (5) Recent history of immunosuppressant use; (6) Pregnant women; and (7) Incomplete or missing follow-up data.

### Data collection

Patients received conventional PD solutions (Dianeal 1.5, 2.5%, or 4.25% glucose), with 3–5 exchanges per day. All patients were followed for up to 5 years from the start of treatment until death or the endpoint. Baseline demographic data were collected within the first 1–3 months after starting PD treatment, including age, gender, history of hypertension, cardiovascular disease (CVD), remission time, cause of ESRD and diabetes, body mass index (BMI), neutrophils, white blood cells, urinary microalbumin, total protein, fibrinogen, creatinine, and blood urea nitrogen. Each patient was followed up at least quarterly for physical examinations and laboratory tests.

### Study definitions

For PD patients, remission time refers to the period during which symptoms of dialysis-related complications (such as peritonitis) are alleviated to an acceptable level or completely disappear under specific treatment. In our study, we defined it as the time when symptoms of peritonitis (such as abdominal pain, fever, and cloudy dialysis effluent) disappear, and the white blood cell count in the dialysis effluent returns to normal after receiving antibiotics or other treatments. Our evaluation criteria include the reduction or disappearance of peritonitis symptoms, the restoration of dialysis effluent clarity, and laboratory tests showing a white blood cell count in the effluent of less than 100/μL. Meanwhile, we enrolled patients who had been receiving PD treatment for at least three consecutive months, and the recorded age is the age at the time of evaluation. Cause of ESRD in the enrolled patients was based on clinical history and manifestations, laboratory findings (urinalysis, 24 h urine protein quantification, serum albumin, lipid profile, renal function tests), imaging studies, and specific serological markers (anti-PLA2R, anti-THSD7A antibodies). AFR is calculated by dividing serum albumin levels by plasma fibrinogen levels. CVD is defined as a history of myocardial infarction, coronary artery bypass grafting, heart failure, atherosclerotic heart disease, or stroke. Patients with a history of type 1 or type 2 diabetes and/or current use of insulin or oral hypoglycemic agents were classified as having diabetes. Hypertension is defined as taking antihypertensive medication or having a blood pressure ≥ 140/90 mmHg on two separate measurements.

### Patient grouping and statistical analysis

We used the “survminer” package in R to determine the optimal cutoff point for AFR in the cohort. The log-rank test and Kaplan–Meier (K-M) curves were used to evaluate survival differences between the low-AFR and high-AFR groups. The “randomForestSRC” package was used for random survival forest analysis to rank the importance of all factors. The prognostic ability of the new model and individual factors was compared using the concordance index (c-index). Univariate and multivariate Cox regression analyses were performed for OS in the entire cohort. Normally distributed continuous variables are expressed as mean ± standard deviation (SD) and compared using Student’s *t*-test. Non-normally distributed continuous variables are presented as median with interquartile range (IQR) and compared using the Mann–Whitney U test. Categorical variables are expressed as percentages and compared using the chi-square test or Fisher’s exact test. Two-sided *p*-values <0.05 were considered statistically significant. Statistical analyses were conducted using SPSS (version 25.0) and R software (version 3.6.1).

### Construction and validation of the prognostic prediction model nomogram

Factors that reached statistical significance in univariate Cox regression analysis were included in the model to construct the prognostic prediction model nomogram. The performance of the nomogram was evaluated using receiver operating characteristic (ROC) curves (using the “pROC” package in R), bootstrapped c-index (using the “rms” package in R), and calibration plots (using the “rms” package in R). The benefit curve of the model was constructed using the “ggDCA” package.

## Results

### Baseline characteristics of patients

A total of 148 PD patients were included in the study. The mean age was 53.2 ± 13.9 years old, with 91 males (61.4%). Hypertension was present in 66 patients (44.6%), diabetes in 33 patients (22.3%), and a history of CVD in 7 patients (4.7%). With regards to the cause of ESRD, only 26.3% of the patients underwent renal biopsy, while 73.7% received a diagnosis based on clinical and laboratory data. Hypertensive nephropathy accounted for 39.2% of cases, primary glomerula disease for 33.8%, including membranous nephropathy, IgA nephropathy, and focal segmental glomerulosclerosis, diabetic nephropathy for 21.6% and polycystic kidney disease for 5.4%. Baseline characteristics of all enrolled patients (*n* = 148) can be seen in Table 1. The optimal AFR cutoff value for OS was determined to be 9.06 using the “survminer” package. Patients were subsequently divided into two groups based on this cutoff value. Patients in the low-AFR group were older, had higher rates of hypertension and diabetes, higher levels of blood urea nitrogen, total protein, urinary microalbumin, globulin, and fibrinogen, but lower levels of albumin and total urine output over 24 h. Baseline clinical characteristics of all patients according to low and high AFR groups are listed in Table 2.

**Table 1:** Baseline characteristics of all enrolled patients (*n* = 148).

Relevant parameters	
Age (years old)^b^	53.2 ± 13.9
Gender (Male)^a^	91 (61.4%)
Cause of ESRD^a^	
Primary Glomerular Disease	50 (33.8%)
Stage II or higher Membranous Nephropathy	42
IgA Nephropathy	5
Focal Segmental Glomerulosclerosis	3
Diabetic Nephropathy	32 (21.6%)
Hypertensive Nephropathy	58 (39.2%)
Polycystic Kidney Disease	8 (5.4%)
Blood Urea Nitrogen (BUN) (mmol/L)^c^	15.1 (12.7–29.9)
Ferritin (ng/mL)^c^	373.1 (162.0–589.7)
24-Hour urine total protein (mg)^c^	5,024 (748.8–11,753)
24-Hour Urine Volume^c^	1,600 (500–2,700)
Cholesterol^c^	7.62 (0.6–14.99)
Triglycerides^c^	1.73 (3.89–12.76)
HDL^c^	1.44 (0.68–2.49)
LDL^c^	5.35 (1.6–10.23)
Baseline Comorbidities^a^	
Hypertension	66 (44.6%)
Diabetes	33 (22.3%)
CVD	7 (4.7%)
Smoke^a^	59 (39.9%)
Mortality	37 (25%)

HDL, high-density lipoprotein; LDL, low-density lipoprotein; ESRD, end-stage renal disease; CVD, cardiovascular disease.

a Values (percentage).

b Mean ± standard deviation.

c Median (range).

**Table 2:** Baseline characteristics based on AFR.

Variable	AFR		*p* value
	≤9.06	>9.06	
Number of patients	30	118	
Age	66.0 (50.0–72.0)	52.0 (42.0–62.0)	<0.001
Blood Urea Nitrogen (BUN) (mmol/L)	16.7 (16.5–24.6)	14.6 (14.1–16.8)	<0.001
Ferritin (ng/mL)	355.0 (280.0–452.6)	380.0 (289.5–446.8)	0.625
White Blood Cells (10^9/L)	5.6 (4.4–6.6)	6.2 (5.0–7.1)	0.009
Hemoglobin (g/L)	128.5 (113.0–137.0)	132.0 (119.0–142.0)	0.544
Platelets (10^9/L)	226.0 (162.0–257.0)	237.0 (202.0–285.0)	0.260
Preoperative Neutrophil Ratio (%)	47.1 (39.6–67.8)	55.5 (50.8–62.6)	0.027
Urine Microalbumin (mg/L)	643.3 (382.9–720.1)	271.1 (157.7–441.0)	<0.001
Total Protein	8723.0 (5041.0–14318.0)	5078.0 (3111.0–6841.0)	<0.001
24-Hour Urine Total Protein (mg)	8041.3 (4459.2–10582.8)	4362.4 (2982.5–6070.0)	<0.001
Urea Nitrogen	16.7 (16.5–24.6)	14.6 (14.1 to 16.8)	<0.001
24-Hour Urine Volume	1250.0 (1000.0–1600.0)	1700.0 (1200.0–2200.0)	0.012
Creatinine (μmol/L)	1083.7 (932.6–1523.6)	748.5 (603.7–1155.2)	<0.001
Cholesterol	7.7 (6.3–12.2)	7.5 (5.6–9.4)	0.127
Triglycerides	1.8 (1.5–2.4)	1.6 (1.2–3.4)	0.335
HDL	1.4 (1.2–1.8)	1.4 (1.1–1.8)	0.713
LDL	5.7 (3.2–8.5)	5.2 (2.9–6.3)	0.040
Remission Time	31.8 (29.8–35.5)	24.1 (19.0–27.5)	<0.001
Hypertension	13 (0.0–1.0)	53 (0.0–1.0)	0.879
Diabetes	13 (0.0–1.0)	20 (0.0–1.0)	0.001
CVD	3 (0.0–1.0)	4 (0.0–1.0)	0.298
Smoke	12 (0.0–1.0)	47 (0.0–1.0)	0.981

HDL, high-density lipoprotein; LDL, low-density lipoprotein; CVD, cardiovascular disease.

### Prognostic value of AFR on mortality

**Figure 1:**
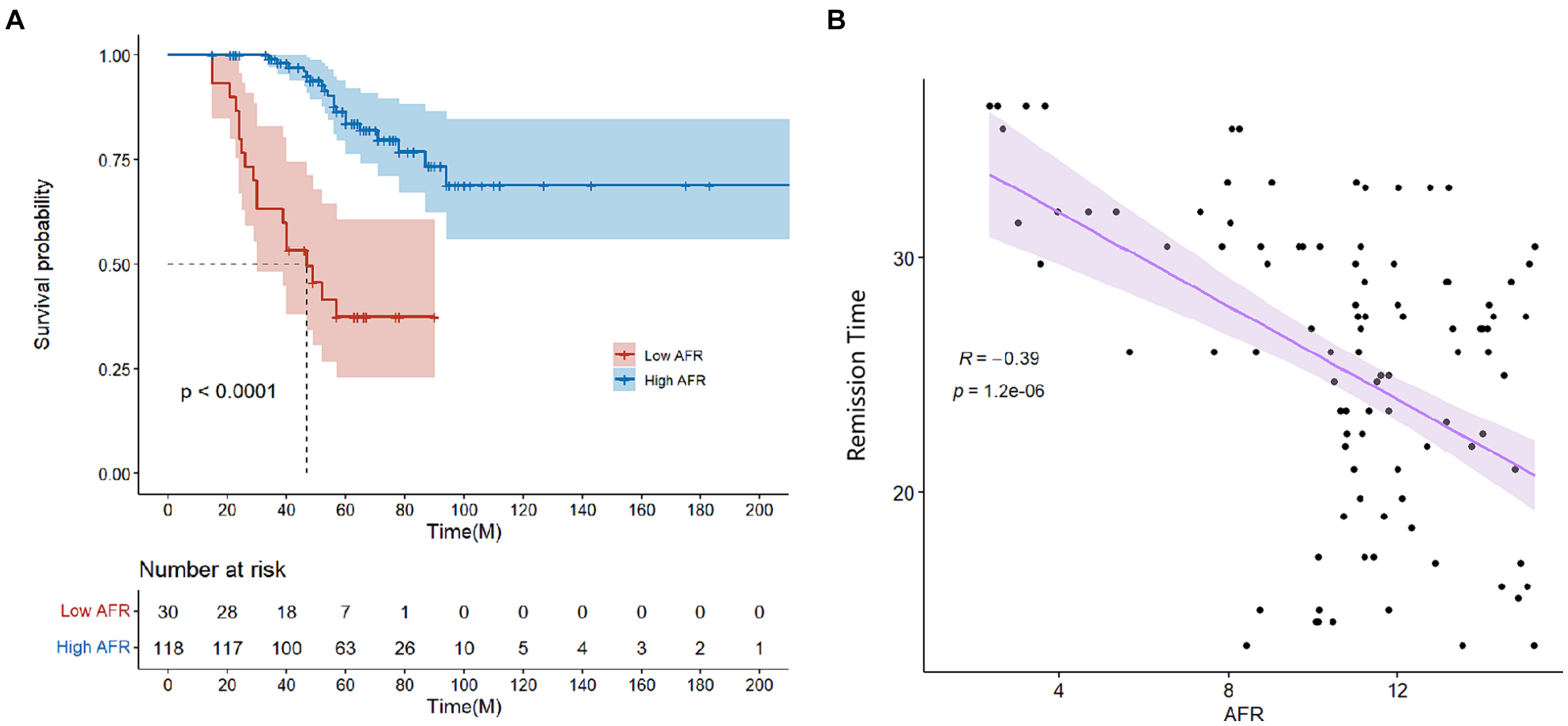
**(A)** Survival curves for patients grouped by high and low AFR (≤9.06 vs. >9.06). **(B)** Correlation between AFR and remission time. AFR, albumin-to-fibrinogen ratio.

**Figure 2:**
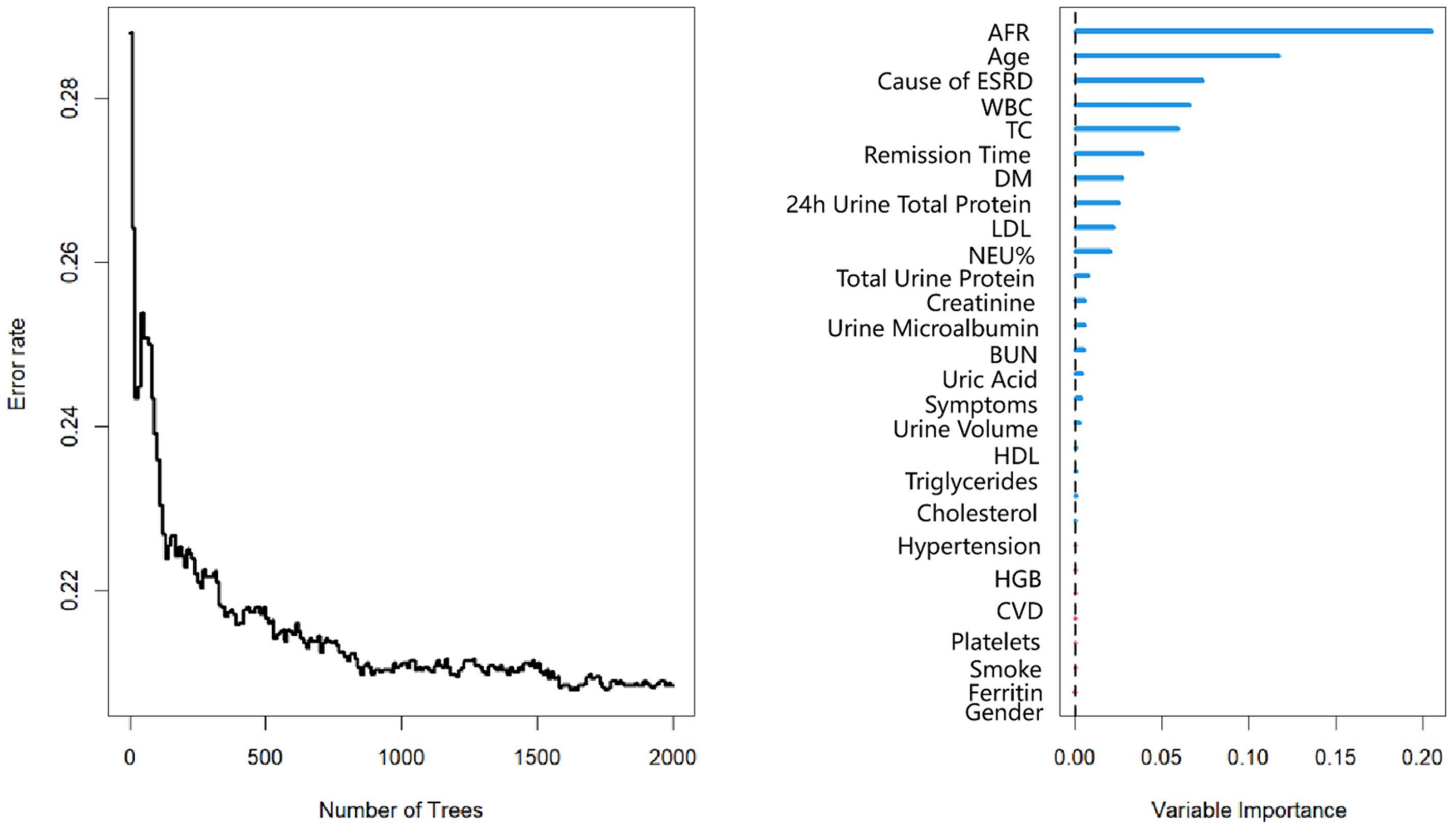
Random survival forest plot for PD patients. AFR, albumin-to-fibrinogen ratio. HDL, high-density lipoprotein; LDL, low-density lipoprotein; CVD, cardiovascular disease. DM, diabetes mellitus; TC, total cholesterol; BUN, blood urea nitrogen; HGB, hemoglobin; WBC, white blood cell; NEU%, neutrophil percentage.

The mean follow-up period was 60.9 months. 25 patients were followed up for 3 years, 85 patients were followed up for 5 years, and 125 patients were followed up for 7 years. At the end of follow-up, 37 deaths were recorded. Kaplan–Meier survival analysis and log-rank tests were used to determine the association between AFR and all-cause mortality. Our results indicated that lower AFR was significantly associated with reduced OS (Figure 1A). Our research found that AFR was closely correlated with remission time, with higher AFR levels associated with longer remission times (*r* = −0.39, *p* < 0.001) (Figure 1B). Furthermore, multivariate Cox regression analysis revealed that low AFR was independently associated with decreased OS (HR 2.39, 95% CI 1.74–3.79, *p* < 0.001). Age (HR 1.04, 95% CI 1.00–1.08, *p* = 0.034) and cause of ESRD (HR 2.76, 95% CI 1.05–7.26, *p* = 0.040) were also identified as independent risk factors for mortality (Tables 3, 4; Figure 2). To further identify clinically significant prognostic factors, we employed a random survival forest algorithm to analyze the importance of all factors, revealing that AFR, age, and cause of ESRD ranked as the top three most important factors (Figure 2), consistent with previous findings.

**Table 3:** Univariate and multivariate cox regression analysis predicting OS in the cohort of PD patients.

Variable	Univariate Analysis			Multivariate Analysis		
	OR	95%CI	*p*	OR	95%CI	*p*
Gender (Male/Female)	1.85	0.87–3.92	0.109			
Age	1.08	1.05–1.11	<0.001	1.06	1.03–1.10	<0.001
Ferritin (ng/mL)	1.00	1.00–1.00	0.324			
Cause of ESRD	2.88	1.85–4.47	<0.001	3.96	1.85–4.47	<0.001
Hemoglobin (g/L)	1.01	0.99–1.03	0.261			
Preoperative Neutrophil Ratio (%)	0.984	0.965–1.003	0.097			
Total Protein	1.00	1.00–1.00	0.189			
24-Hour Urine Total Protein	1.00	1.00–1.00	0.103			
Urea Nitrogen	1.08	1.01–1.16	0.034	0.96	0.88–1.05	0.413
Symptoms	1.67	0.80–1.94	0.018	1.24	1.09–2.56	0.334
24-Hour Urine Volume	1.00	1.00–1.00	0.473			
Creatinine (μmol/L)	1.00	1.00–1.01	0.330			
Cholesterol	1.06	0.93–1.21	0.398			
Triglycerides	0.88	0.70–1.09	0.237			
HDL	0.99	0.49–2.01	0.978			
LDL	1.10	0.96–1.27	0.179			
Hypertension	0.98	0.51–1.88	0.949			
AFR	0.16	0.08–0.30	<0.001	0.15	0.07–0.32	<0.001
DM	3.42	1.79–6.54	<0.001	0.85	0.40–1.84	0.686
CVD	1.40	0.43–4.59	0.576			
Smoke	0.82	0.42–1.59	0.552			

HDL, high-density lipoprotein; LDL, low-density lipoprotein; ESRD, end-stage renal disease; CVD, cardiovascular disease. DM, diabetes mellitus; AFR, albumin-to-fibrinogen ratio.

**Table 4:** C-index of the prognostic prediction model.

Endpoint	Variable	C index	95%CI
OS	Nomogram Prognostic Model	0.858	0.794–0.921
	Age	0.750	0.673–0.825
	AFR	0.728	0.649–0.803
	Cause of ESRD	0.676	0.580–0.771

AFR, albumin-to-fibrinogen ratio.

### Construction and evaluation of the prognostic model

**Figure 3:**
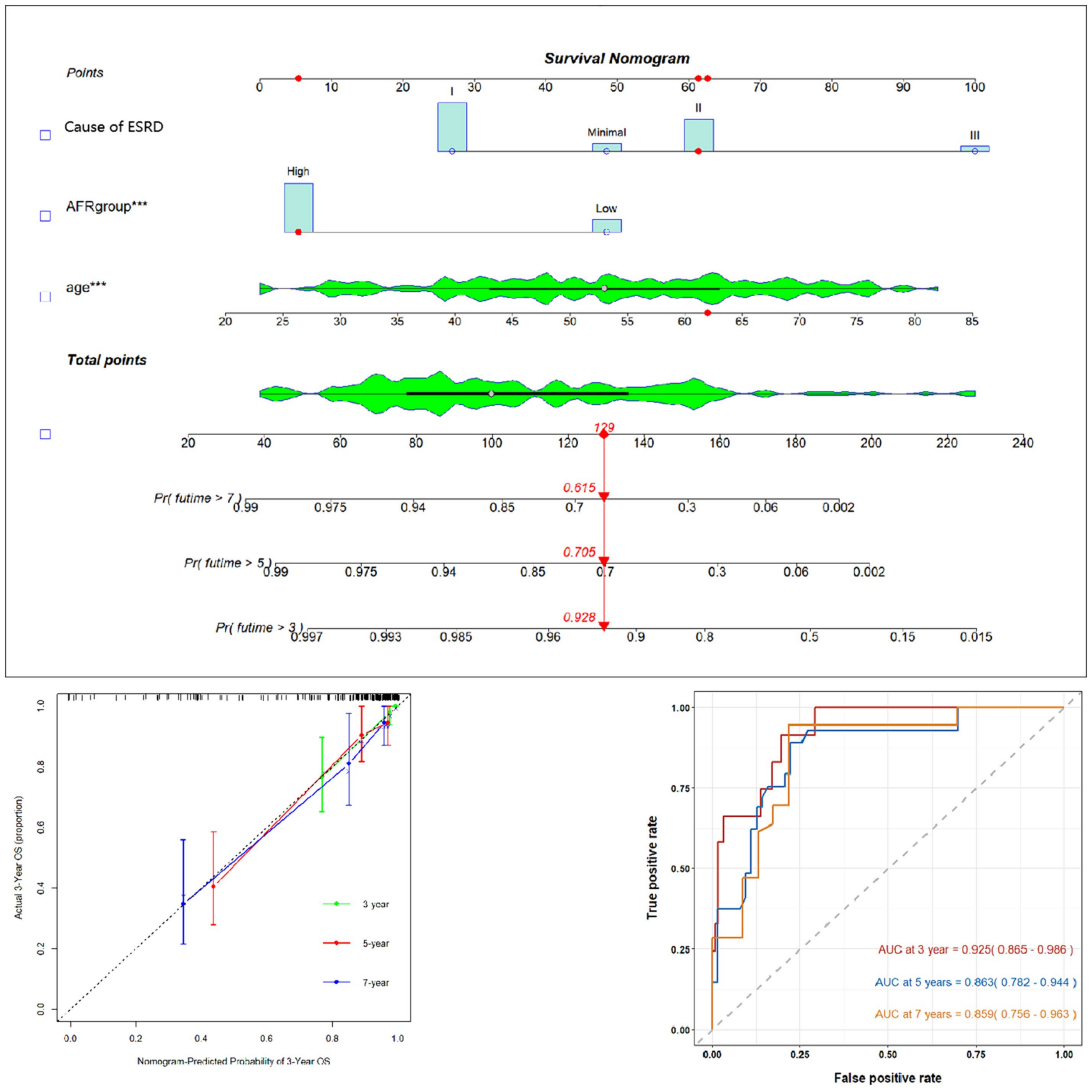
Prognostic prediction model nomogram and calibration curve based on age, AFR, and cause of ESRD. AFR, albumin-to-fibrinogen ratio.

Using AFR, age, and cause of ESRD, we constructed a prognostic nomogram (Figure 3) to assess the 3-year, 5-year, and 7-year survival probabilities of patients. For instance, a patient with stage II membranous pathology, a high AFR, and an age of 62 years had survival probabilities of 92.8, 70.5, and 61.5% at 3, 5, and 7 years, respectively. To validate the reliability of the model, we used calibration curves and ROC curves for evaluation. The 3-year, 5-year, and 7-year calibration curves were close to the diagonal line, indicating that the predicted probabilities were close to the actual occurrences, demonstrating the model’s predictive accuracy. The areas under the ROC curves (AUC) for 3, 5, and 7 years were 0.925, 0.863, and 0.859, respectively. Although the AUC decreased slightly over time, it still indicated high predictive accuracy for the prognostic model.

### Benefit curves construction

**Figure 4:**
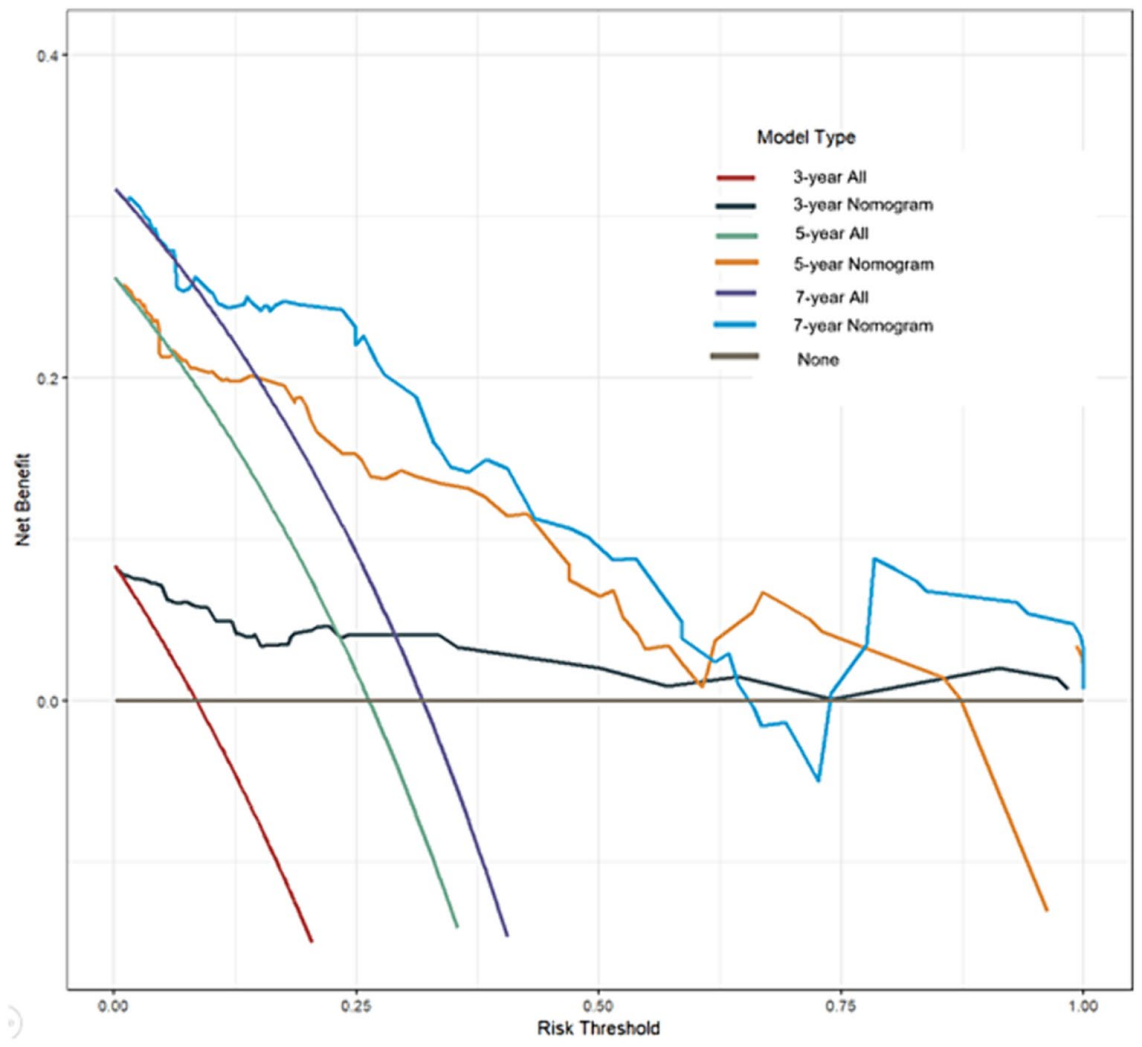
Benefit curves at different time points.

Benefit curves were used to evaluate the model’s performance in prediction. The benefit curves help us understand the model’s performance at different thresholds, as shown in Figure 4. The x-axis represents the threshold, and the y-axis represents cumulative benefits, with each curve representing 3-year, 5-year, and 7-year outcomes. The decision curve analysis indicated that, for the 3-year mark, when the model threshold was set between 0.1 and 98.1%, the decision curve was above the None line and the All line. For the 5-year mark, the threshold range was 0.8 to 87.3%, and for the 7-year mark, the range was 1.2 to 64.3%. This demonstrates that the model has clinical utility within these ranges. Additionally, the overall c-index for the model was 0.858 (0.794–0.921), compared to 0.750 (0.673–0.825) for age alone, 0.728 (0.649–0.803) for AFR alone, and 0.676 (0.580–0.771) for cause of ESRD alone. This indicates that the predictive model combining age, AFR, and pathology has a higher predictive performance than any single factor.

### Development and validation of the prognostic prediction model nomogram

An OS prognostic prediction model nomogram was constructed using the entire cohort data. The final nomogram, incorporating age, cause of ESRD, and preoperative AFR, along with its calibration curve, is shown in Figure 3. These independent risk factors were used to predict the probability of OS.

## Discussion

Chronic kidney disease (CKD) remains a major global public health concern, with its prevalence continually rising, leading to significant morbidity and mortality. Peritoneal dialysis (PD) has become a crucial and widely utilized treatment for patients with CKD, offering numerous advantages over hemodialysis (HD), such as greater patient autonomy, improved quality of life, and cost-effectiveness (13). Despite these benefits, PD patients continue to face high all-cause mortality rates, highlighting the critical need for identifying sensitive prognostic biomarkers and implementing effective interventions to improve long-term survival outcomes.

Serum albumin levels are traditionally used as indicators of nutritional status and infection (14, 15). The kidney’s handling of albumin, through its filtration in the glomeruli and reabsorption in the renal tubules, plays a vital role in maintaining renal function. Research by Yamada et al. demonstrated that lower serum albumin levels in PD patients are associated with an increased risk of residual renal function loss, ultimately deteriorating the patient’s overall condition and leading to death (16). Multiple studies have consistently indicated that lower serum albumin levels correlate with decreased survival rates in PD patients (17). When average albumin levels and serum albumin achievement rates are used as predictive variables, higher serum albumin levels in patients undergoing long-term PD are associated with reduced all-cause mortality (18). This suggests that hypoalbuminemia is a significant risk factor for both early and late mortality in this population.

Recent research has focused on the relationship between serum albumin levels and pro-inflammatory cytokines in PD patients. Inflammatory cytokines such as TNF-α and IL-6 inhibit albumin synthesis, indicating that hypoalbuminemia in PD patients may be more attributable to systemic inflammation rather than malnutrition (19, 20). This inflammatory state significantly contributes to the increased mortality observed in PD patients with low serum albumin levels. Additionally, plasma fibrinogen levels, which respond to inflammation and coagulation activation, are consistently elevated in CKD patients. The loss of albumin in peritoneal dialysis fluid can lead to the accumulation of free fatty acids in the blood, stimulating hepatic fibrinogen synthesis (21). Moreover, prolonged exposure to glucose-based dialysis fluids can induce severe metabolic syndrome, resulting in endothelial dysfunction, heightened inflammation, and a prothrombotic state (22). Consequently, PD patients are more prone to thrombotic events. A review of the literature indicates conflicting results regarding whether elevated plasma fibrinogen is a risk factor for mortality in CKD and PD patients, suggesting a complex relationship between fibrinogen levels and patient outcomes (23, 24).

Given these complexities, relying on a single biological marker to predict the prognosis of PD patients may be insufficient. Our study found that the albumin-to-fibrinogen ratio (AFR), compared to individual serum albumin or plasma fibrinogen levels, provides a superior predictive value for all-cause mortality risk in PD patients. An AFR ≤ 9.06 was identified as a risk factor for lower survival, with overall survival (OS) in the high-AFR group (> 9.06) being significantly higher than in the low-AFR group (≤ 9.06). Patients in the low-AFR group were typically older (*p* < 0.01) and had higher levels of blood urea nitrogen (*p* < 0.001), suggesting that low AFR is associated with complex malnutrition and metabolic dysfunction, which may contribute to poor prognosis. Simultaneously, the outcomes of this study align with the findings of Professor Claps’ research. Their multicenter retrospective analysis revealed that a preoperative low albumin-to-fibrinogen ratio (AFR) serves as a prognostic biomarker for poorer time to progression (TTP), overall survival (OS), and cancer-specific survival (CSS). Additionally, it is independently correlated with adverse pathological features in bladder cancer (BC) patients undergoing radical cystectomy (RC). They further recommend that patients with low AFR should be considered for neoadjuvant therapy, underscoring the pivotal role of AFR as a novel biomarker in the context of various immunometabolic diseases (25). To improve the prognostic assessment of PD patients, we developed an OS nomogram incorporating AFR, age, and cause of ESRD. The results indicated that this nomogram accurately predicts the prognosis of PD patients, as evidenced by the AUC and c-index values. Our nomogram outperformed individual predictors such as age, cause of ESRD, and AFR alone, further validating its reliability and utility in clinical practice.

Age has been consistently shown to influence clinical outcomes and mortality in PD patients. Our findings align with those of Sakaci et al., who reported higher mortality rates among older PD patients, with lower albumin levels often exacerbating this risk (26). Diabetes mellitus (DM), a prevalent metabolic disease, also plays a significant role in the prognosis of PD patients. The pathogenesis of type II DM is associated with insulin resistance (IR) and β-cell dysfunction (27). PD involves higher glucose loads compared to HD due to the routine use of glucose-based solutions, leading to rapid absorption and metabolism of glucose following peritoneal absorption (28, 29). The molecular size of glucose is quite small, at 180 Da. Therefore, it is rapidly absorbed by the peritoneum and metabolized after entering the bloodstream. Due to the increased risk of hypoglycemia, HD patients rarely use glucose-free dialysate. Although glucose is one of the components of HD solutions, the glucose load in HD patients is much lower than that in PD patients (30). Due to the rapid uptake of glucose, especially when the dialysate remains in the peritoneal cavity for a prolonged period, the ultrafiltration capacity decreases or even stops. This phenomenon can be counteracted by adding a higher glucose concentration to the PD solution, leading to a steeper osmotic gradient but also resulting in higher systemic glucose absorption. This increased glucose exposure can lead to decreased ultrafiltration capacity and metabolic complications (31). Studies have shown that approximately 25% of PD patients develop new-onset hyperglycemia, even with standard glucose concentrations (29). Previous studies have indicated that diabetes mellitus (DM) is associated with poorer prognosis in PD patients (32). Our study identified a history of DM as a risk factor for poor prognosis in PD patients, highlighting the need for refined management of blood glucose levels and vigilant monitoring for new-onset DM.

The interplay between albumin and fibrinogen levels in the context of inflammation and malnutrition further complicates the prognostic landscape for PD patients. Albumin, as a negative acute-phase reactant, tends to decrease during inflammatory states, whereas fibrinogen, a positive acute-phase reactant, increases. This opposing behavior highlights the importance of considering both markers in tandem, as AFR provides a more comprehensive assessment of a patient’s inflammatory and nutritional status. The inflammatory milieu in PD patients not only accelerates vascular damage but also impairs the body’s ability to maintain homeostasis, leading to adverse outcomes.

Furthermore, our study’s findings underscore the importance of integrating multifaceted biomarkers into prognostic models. The use of AFR as a combined biomarker leverages the individual prognostic strengths of albumin and fibrinogen, offering a more robust prediction model. The development of the AFR-based nomogram, which includes age and cause of ESRD, represents a significant advancement in personalized medicine for PD patients. The nomogram’s superior predictive accuracy, as demonstrated by the AUC and c-index values, highlights its potential utility in clinical decision-making, enabling healthcare providers to stratify patients more effectively and tailor interventions accordingly.

The retrospective nature and single-center scope of our study, along with the relatively small sample size, present limitations that must be addressed in future research. Prospective, multicenter studies are necessary to validate the prognostic value of AFR in a broader population of PD patients. Meanwhile, a significant limitation in our study is that the cause of ESRD was based on clinical and laboratory data, without a kidney biopsy, in two thirds of enrolled patients (73.7%). This limitation is particularly relevant, as the cause of ESRD was one of the three elements that were used to build the nomogram. However, when histological data were not available, causes of ESRD were established on a robust framework of clinical and laboratory data. We plan to address this limitation in future research through multicenter prospective studies where more comprehensive diagnostic strategies, including renal biopsies when feasible, can be integrated to validate our current findings. Such studies would enhance the generalizability of our findings and provide a more comprehensive understanding of the role of AFR in predicting patient outcomes. Additionally, investigating the underlying mechanisms driving the relationship between AFR and mortality in PD patients could yield valuable insights, potentially uncovering novel therapeutic targets to improve patient outcomes. In conclusion, the albumin-to-fibrinogen ratio (AFR) is a promising prognostic biomarker for PD patients. Our study demonstrated that AFR is a better predictor of all-cause mortality compared to individual serum albumin or plasma fibrinogen levels. The constructed nomogram, incorporating AFR, age, and cause of ESRD, provides an accurate tool for predicting the prognosis of PD patients, thereby aiding clinicians in making informed decisions to enhance patient care. Further research is warranted to validate these findings and explore additional biomarkers that may improve prognostic accuracy in this patient population.

## Conclusion

In summary, AFR is a potential prognostic biomarker for PD patients. A nomogram incorporating AFR can provide accurate predictions of OS in PD patients, aiding clinicians in making better-informed decisions.

## Data Availability

The raw data supporting the conclusions of this article will be made available by the authors, without undue reservation.
